# Classification of monthly tidal envelopes in mixed tide regimes

**DOI:** 10.1038/s41598-023-31657-x

**Published:** 2023-03-23

**Authors:** Do-Seong Byun, Deirdre E. Hart, Sangil Kim, Jeongmin Ha

**Affiliations:** 1 Ocean Research Division, Korea Hydrographic and Oceanographic Agency, Busan, 49111 Republic of Korea; 2 Faculty of Science, University of Canterbury, Private Bag 4800, Christchurch, New Zealand; 3 Department of Mathematics, Pusan National University, Busan, 46241 Republic of Korea; 4 Finance, Fishery Manufacture Industrial Mathematics Center on Big Data, Pusan National University, Busan, 46241 Republic of Korea

**Keywords:** Ocean sciences, Natural hazards

## Abstract

Coastal inundation is increasing globally. Changes in tidal water levels contribute to flood risk alongside rain and sea storm events. Unlike the latter, temporal variations in tides may be predicted and their patterns analyzed many years in advance. This paper explains two novel methods for characterizing monthly scale patterns in tidal water level variation: one simple qualitative method with restricted applicability; and another more complex quantitative method with global applicability to areas characterized by mixed, mainly semidiurnal and mixed, mainly diurnal tide regimes (~ 65% of global oceans). We reveal that in some areas tidal high and low waters are balanced in near symmetrical patterns, while elsewhere tides are skewed towards upper or lower tidal height envelopes. Areas characterized by tidal patterns skewed towards upper envelopes are at heightened risk of extreme event inundations during certain periods each year, event scale risks that will increase with climate changes. Those skewed towards lower tidal envelopes are prone to frequent flooding and are potentially at greater risk of chronic inundation with ongoing mean sea level rise. Our findings and the novel tidal pattern classification approaches offered contribute to understanding the time varying nature of tidal contributions to coastal inundation risks.

## Introduction

Understanding temporal patterns in tidal heights (tides) is central to many coastal hazard and development activities^1–3^. Extreme inundation and nuisance flooding are growing challenges worldwide, with events and impacts projected to increase with ongoing climate changes^3–5^. These hazards occur in low-lying settlements affected by storm surges, plus significant rainfall and/or river flows, coincident with higher-than-average tides^3–9^. Both the storm surge and freshwater components of such events operate over synoptic timescales^10^ and are difficult to predict much in advance. The tide components of such events are highly predictable, so can be used to forecast potential flood alerts^11^—periods with sustained, anomalously high tides predisposing low-lying areas to inundation should storms occur. In this context it is useful to consider ways of characterizing variations in tidal form patterns from place to place.

### Tidal form classification background

The daily tidal form factor *F*^12,13^ has been successfully used decades to compare tide characteristics^14^. Elegantly simple, *F* reflects the ratio of the sums of the 2 major semidiurnal (M_2_, S_2_) versus diurnal (K_1_, O_1_) tidal constituents’ amplitudes: $\frac{{K}_{1}+{O}_{1}}{{M}_{2}+{S}_{2}}$. Though important for comparing daily tides from place to place, with its terms semidiurnal, diurnal and mixed in everyday usage, the descriptive power of *F* is confined to *daily* tidal forms. Tides exhibit additional variation at monthly timescales, including spring/ neap, perigean/ apogean, and mixed cycles. As a simple amplitude ratio, *F* cannot be used to distinguish variations in tides occurring at greater than daily timescales, including asymmetries between high and low waters that have implications for inundation^2,5,15^. This is because, in addition to amplitudes, tidal constituent phase lags also contribute to monthly patterns and asymmetries in tidal forms.

Similar to the original development of *F*, a monthly tidal envelope form (TEF) factor *E* was developed for semidiurnal regimes using sea level records from New Zealand^14^. The present paper extends the classification of monthly TEF factors to mixed, mainly semidiurnal (0.25 < *F* < 1.5) and mixed, mainly diurnal (1.5 < *F* < 3) tidal regimes (collectively ‘mixed tides’ or ‘mixed tidal regimes’). To achieve this, the key characteristics of monthly TEFs in mixed regimes were explored and employed to develop a simple TEF factor applicable to certain mixed regimes based on phase lag differences between three major tidal constituents. A new, generalized TEF classification approach was also developed using tidal species modulation methods^16,17^. While more complex and needing additional work, this latter approach gives quantitative results and is applicable in any mixed tide regime.

### Characteristics of monthly tidal patterns in mixed tidal regimes

**Figure 1:**
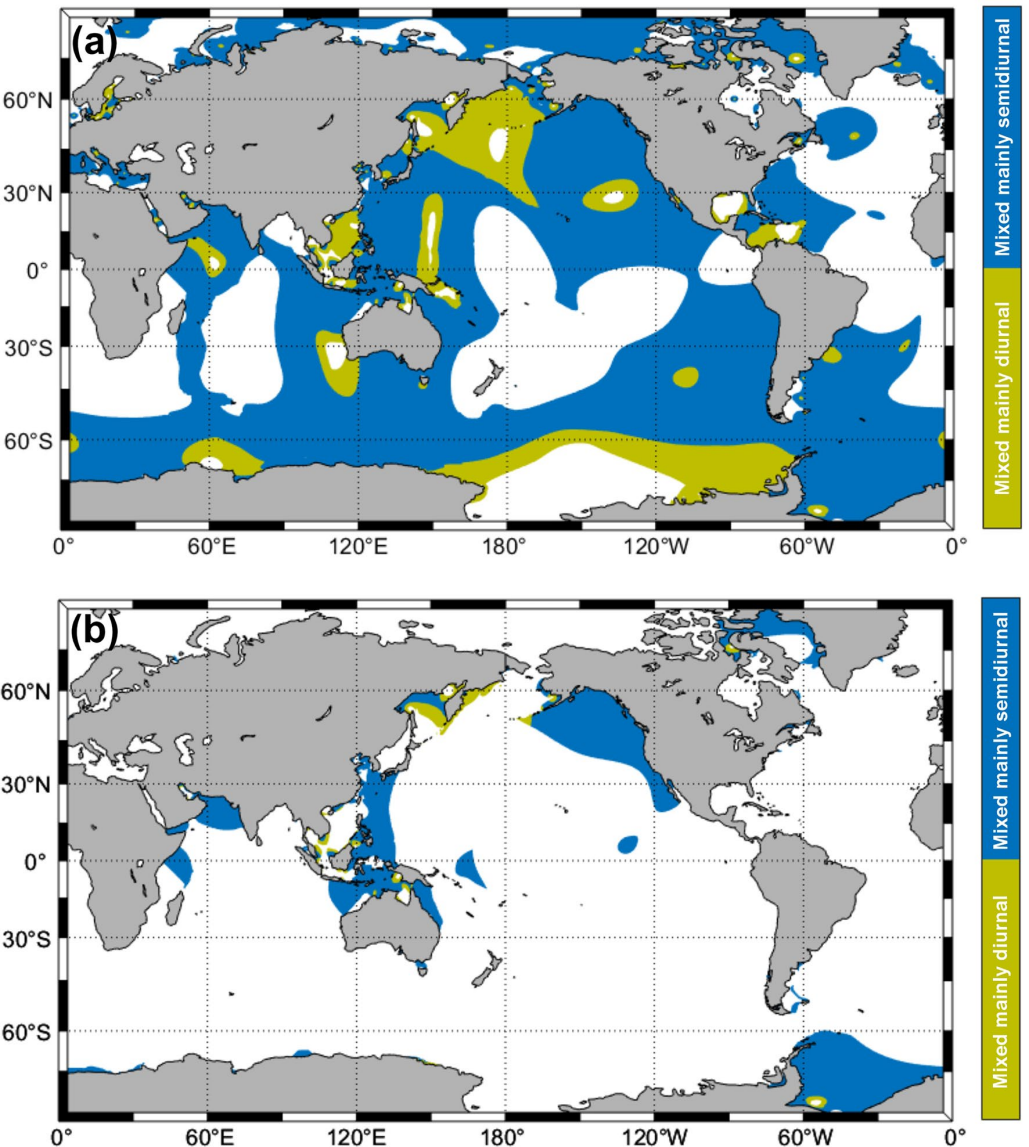
(**a**) Global distribution of mixed, mainly semidiurnal (0.25 < *F* < 1.5) and mixed, mainly diurnal (1.5 < *F* < 3.0) tides (classified using *F*); and (**b**) of mixed tidal regimes with tidal ranges > 2 m (calculated as twice the sum of the M_2_, S_2_, K_1_ and O_1_ amplitudes). Maps were derived from the FES2014 tidal harmonic constants dataset.

Over 64% of Earth’s oceans are occupied by mixed tidal regimes: 56.4% featuring mixed, mainly semidiurnal tides; and 8% featuring mixed, mainly diurnal tides; with the remaining 31.4% occupied by semidiurnal tides and 4.2% occupied by diurnal tides (Fig. 1a). Coastal zones with mixed tide regimes featuring tidal ranges > 2 m are patchily distributed but occur along the coast of every major landmass (Fig. 1b). In the context of inundation, it is important to understand and identify tidal envelope patterns for mixed tidal regimes. In semidiurnal regimes, monthly TEFs tend to exhibit more regular daily forms, with near symmetric upper and lower envelopes^14^. However, in mixed regimes, the combination of semidiurnal and diurnal tides results in changing daily tidal forms throughout each month, including asymmetric high and low waters and changing differences between consecutive highs and consecutive lows. Zelter^18^ explained the diurnal inequality of mixed regimes based on the difference between the phase lags of the M_2_ and (K_1_ + O_1_) tides, with the sum of the angular speeds of the K_1_ (15.0410686° hr^−1^) and O_1_ (13.9430356° hr^−1^) tides equivalent to that of the M_2_ tide (28.9841042° hr^−1^). From the diurnal inequality of tidal form, monthly tidal envelope patterns can be broadly classified as upper or lower tidal envelope dominated (UTE or LTE), or near-symmetric (STE)^19^.

**Figure 2:**
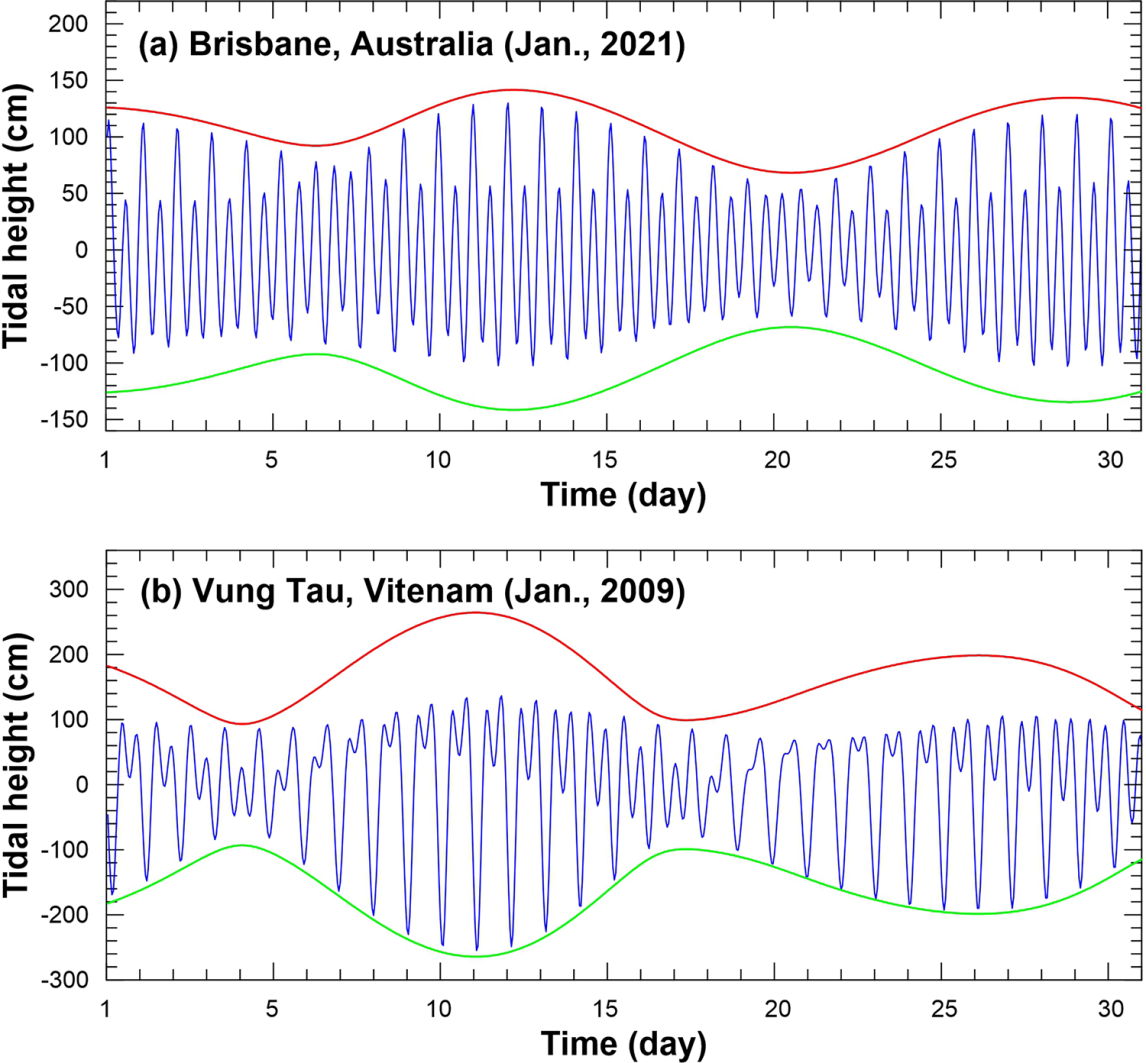
Comparison of different types of tidal pattern asymmetry using observed tidal height time series versus the maximum and minimum tidal envelope curves (green lines) for: (**a**) Brisbane, Australia (January 2021), with its upper tidal envelope dominant regime, and (**b**) Vung Tau, Vietnam (January 2009), with its lower tidal envelope dominant tidal regime, with both sites featuring mixed, mainly semidiurnal tides.

Coastal regimes with UTE dominance (Fig. 2a) are characterized by diurnal inequalities featuring larger differences between consecutive high waters than between consecutive low waters. Such places are prone to inundation when extreme sea storms and/or river floods coincide with spring or spring-perigean tides^2,5,7^. For such places, high inundation risk periods can be forecast in advance to provide communities with warnings to watch for coincident synoptic or river flow conditions likely to result in coastal flooding^11^. With rising mean sea levels and increased storminess forecast as a result of ongoing anthropogenic climate change, such places will likely become more frequently affected by high tide flooding during multi-hazard (e.g. combined tide, storm and/or river flood) events^2,5,7^.

In contrast, coastal regimes with LTE dominance (Fig. 2b) are characterized by diurnal inequalities with larger differences between consecutive low waters. In such environments the consistent high water envelope plus the variable low water envelope can result in chronic flooding of low-lying areas or, when combined with multi-hazards such as sea storms and/or high river flows, this tidal pattern can produce cyclically frequent flooding due to the periodically ineffective terrestrial drainage^3,7,15^. With ongoing mean sea level rise, these LTE dominant environments could be prone to sustained everyday flooding, should the elevation of mean sea levels push tidal operating ranges above efficient drainage and/or land elevation thresholds^3–5^.

## Developing monthly TEF analysis approaches for mixed tide regimes

### E_A_, a qualitative TEF factor derived from phase lag relationships

As explained earlier, three general types of monthly TEF occur in mixed regimes (0.25 < *F* < 3). To investigate the tidal constant conditions resulting in each type, eight simplified tidal envelope generating experiments were conducted. These examined variations in the sum of the tidal species’ modulated amplitudes arising from differences in the diurnal and semidiurnal species’ modulated phase lags.

Five major tidal constituents (K_1_, O_1_, M_2_, S_2_ and N_2_), with their differing tidal phases, were employed in experiments (Table 1). Based on the understanding that TEFs vary with the changing diurnal and semidiurnal tide interactions, we focused on tidal phase lag differences. To simplify experiments, each tidal species was given a single phase-lag value, with the diurnal species phase lag values changing across experiments. Constituent amplitude values were determined based on their Equilibrium Theory amplitude ratios^20^ and an M_2_ amplitude of 100 cm. *F* values were set at 0.75, indicating mixed, mainly semidiurnal regimes.

**Table 1:** Amplitude and phase lag input values for simplified tidal envelope generating experiments.

Experiments	Tidal constants	Diurnal		Semidiurnal			${E}_{A}$	${E}_{M}$	Type
		[s = 1]		[s = 2]					
		K_1_	O_1_	M_2_	S_2_	N_2_			
Cases 1–8	${a}_{i}^{(s)}$[cm]	58	48	100	42	19	–	–	–
Case 1	${g}_{i}^{(s)}$[°]	0	0	0	0	0	1	0.24	UTE
Case 2		30	30	0	0	0	0.5	0.11	UTE dominant
Case 3		45	45	0	0	0	0	0	STE
Case 4		60	60	0	0	0	− 0.5	− 0.11	LTE dominant
Case 5		90	90	0	0	0	− 1	0.24	LTE
Case 6		120	120	0	0	0	− 0.5	− 0.11	LTE dominant
Case 7		135	135	0	0	0	0	0	STE
Case 8		150	150	0	0	0	0.5	0.11	UTE dominant

All simplified experiments represent mixed mainly semidiurnal regimes (tidal form number *F* = 0.75). Amplitude ($${a}_{i}^{(s)}$$) and phase lag ($${g}_{i}^{(s)}$$) values are based on the Equilibrium Theory amplitude ratios between 5 major tidal constituents (K_1_, O_1_, M_2_, S_2_, and N_2_) and $${E}_{A}=\mathrm{cos}({I}_{A})$$ with the diurnal inequality phase lag relationship, $${I}_{A}$$ = ($${{g}_{{K}_{1}}+{g}_{{O}_{1}})-g}_{{M}_{2}}$$. *s* indicates tidal species, while *i* indicates each tidal constituent. $${E}_{M}$$ is the difference between the mean upper tidal envelope and absolute mean lower tidal envelope (see Eq. (9)). UTE is upper tidal envelope (with subcategories ‘UTE’ which has an upper curve that almost perfectly matches the maximum potential upper tidal envelope, and ‘UTE dominant’ which has an upper curve that closely approximates the maximum potential upper tidal envelope) and ‘LTE’ denotes lower tidal envelope (with two equivalent subcategories). STE denotes a near symmetrical tidal envelope.

Results reveal that, when diurnal and semidiurnal tides are in phase, the tidal envelopes produced tend to follow the maximum highs and minimum lows of the semidiurnal ‘pairs’ of high and low waters. Thus, envelope patterns in regimes dominated by M_2_ and K_1_ or O_1_ tides can be identified simply, based on the difference in phase lags ($g$) between these diurnal and semidiurnal constituents, and asymmetries between the upper and lower tidal envelopes can be calculated using the tidal envelope factor (${E}_{A}$) where:

1 $${E}_{A}=\mathrm{cos}\left({I}_{A}\right),$$

with $${I}_{A}$$, the diurnal inequality phase lag relationship, expressed as $${I}_{A}$$ = ($${{g}_{{K}_{1}}+{g}_{{O}_{1}})-g}_{{M}_{2}}$$.

**Figure 3:**
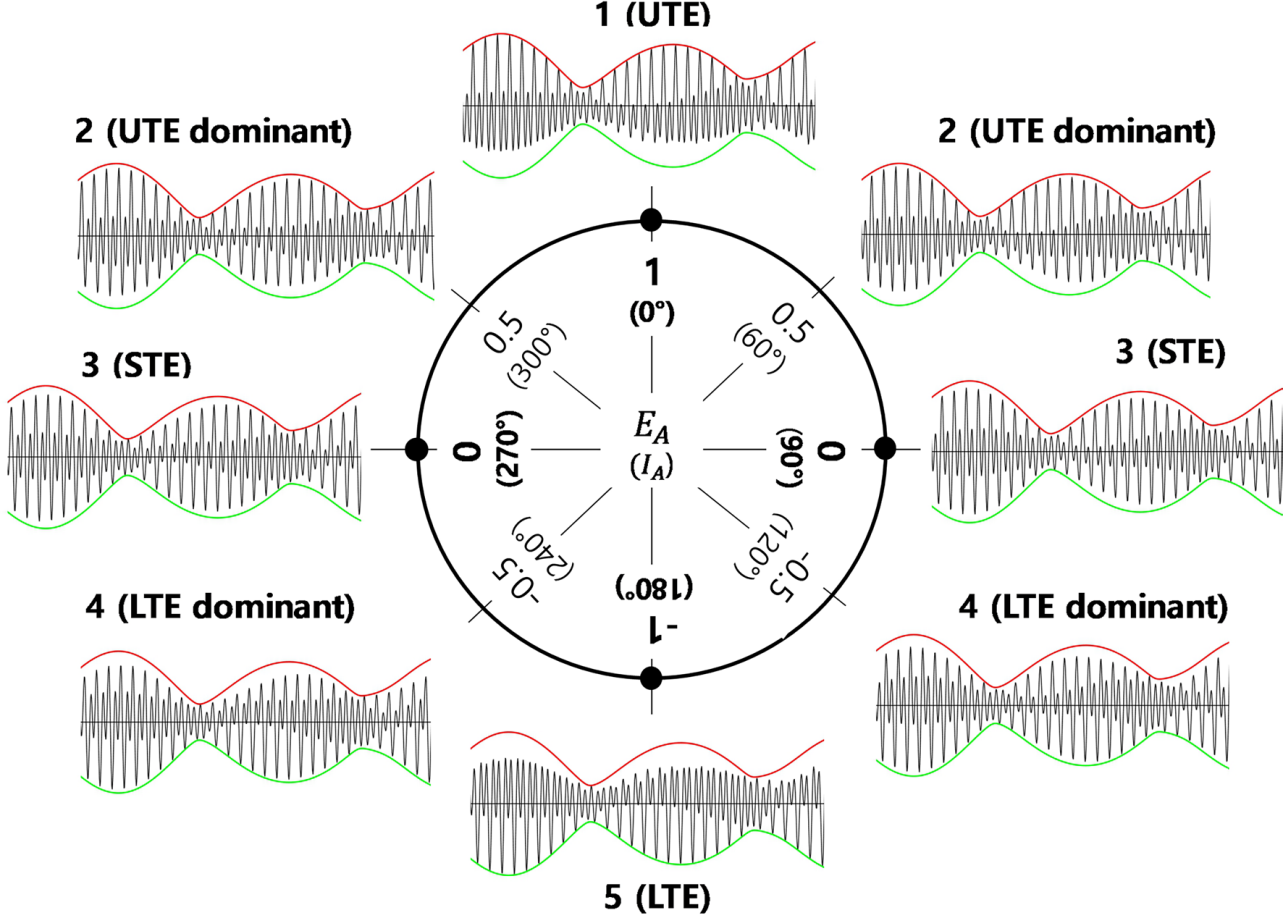
Monthly tidal envelope patterns calculated for a mixed, mainly semidiurnal tidal regime from the tidal amplitude and phase lag values of 5 major tidal constants (Table 1), and classified according to phase lag differences [${E}_{A}=\mathrm{cos}({I}_{E})$, ${I}_{E}={({g}_{{K}_{1}}+{g}_{{O}_{1}})-g}_{{M}_{2}}$]. UTE and LTE denote upper tidal envelope and lower tidal envelope, respectively. STE denotes a near-symmetric tidal envelope. Red and green lines denote the potential maximum upper and lower envelopes (PUE and PLE), calculated from Eqs. (5a), (5b).

As illustrated in Fig. 3, in case 1 where ${E}_{A}=1 \,({I}_{A}=0^\circ$), the curve generated follows the potential maximum upper envelope (PUE) while in Case 5 where ${E}_{A}=-1 ({I}_{A}=180^\circ$) the curve generated follows the potential minimum lower envelope (PLE) (see the next section of this paper for more details on PUE and PLE).

When $-1<{E}_{A}<1 \,(\mathrm{i}.\mathrm{e}. 0^\circ <{I}_{A}<180^\circ$ or $180^\circ <{I}_{A}<360^\circ ),$ the resultant tidal envelopes fail to follow PUEs and PLEs. Tidal envelopes generated in mixed regime settings where the diurnal and semidiurnal species’ interactions are tightly in phase, out of phase, or somewhere between, fail to reach their potential maximum and minimum tidal range envelopes. Here STE conditions (cases 3 and 7, where ${E}_{A}=0,\mathrm{\, and\, }{I}_{A}=90^\circ$ or $270^\circ$) generate the highest tidal ranges, with similar upper and lower envelope patterns. STE conditions can be broadly delimited as those where $-0.18\le {E}_{A}\le 0.18$. When $0.18<{E}_{A}<1\, (\mathrm{i}.\mathrm{e}. 0^\circ <{I}_{A}<79.6^\circ$ or 280.4 $^\circ <{I}_{A}<360^\circ )$ as in Experiment 1 cases 2 and 8, the UTE exceeds the absolute LTE, and is thus labelled UTE dominant. In contrast, when $-1<{E}_{A}<-0.18 (\mathrm{i}.\mathrm{e}., 100.4^\circ <{I}_{A}<180^\circ$ or 180 $^\circ <{I}_{A}<280.4^\circ )$ as in cases 4 and 6, the absolute LTE exceeds the UTE, and this type is thus labelled LTE dominant.

**Figure 4:**
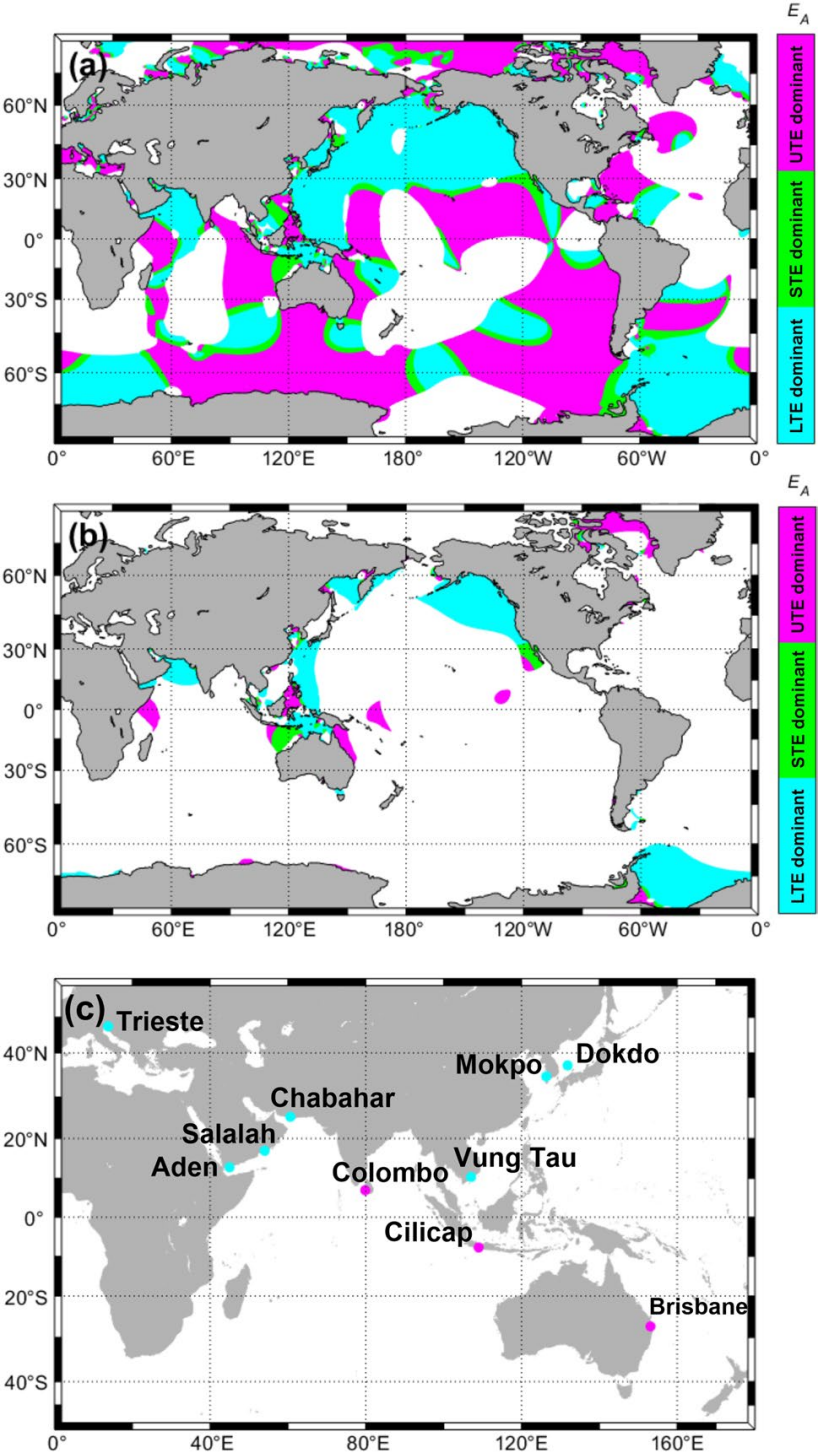
(**a**) Distribution of monthly tidal envelope types throughout the mixed tide regime areas of the globe (0.25 < *F* < 3.0) classified using ${E}_{A}$; (**b**) global distribution of mixed tidal regimes with tidal ranges > 2 m, also classified using ${E}_{A}$; and (**c**) locations of the ten sea level observation sites analyzed in Table 2 (pink and blue dots indicate UTE and LTE dominant locations, respectively). Maps were derived from the FES2014 tidal harmonic constants dataset.

Figure 4 illustrates the global distribution of monthly tidal envelope forms classified according to ${E}_{A}$. Large areas of eastern Asia and western North America are characterized by LTE dominant tidal regimes as well as the Southern Ocean adjacent to the horn of Africa. Much of the central, south-east and south-west Pacific Ocean and most Southern Ocean areas adjacent to the Pacific and Atlantic Oceans are characterized by UTE dominant regimes. Of the areas identified as having asymmetric tides, those with the largest tidal ranges occur in parts of southeastern Asia and the Middle East, and along the west coast of North America (Fig. 4b). In summary, ${E}_{A}$ as defined in Eq. (1) is a straightforward, useful parameter for classifying TEF types in mixed tidal regimes, based on simple manipulation of tidal amplitude and phase lag data from three major tidal harmonic constants. However, ${E}_{A}$ does not offer information on the quantitative similarity between upper and lower tidal envelopes and its accurate application is limited to regimes dominated by the M_2_, K_1_ and O_1_ tides.

### E_M_, A universal, quantitative mixed tide TEF factor

The high and low water heights needed to draw an anytime basic tidal envelope can be extracted from a tidal prediction time series. But this approach lacks the information needed to quantitatively characterize anomalous patterns of variation in highs and lows. Such tidal envelope anomalies can be evaluated via comparison with potential maximum upper and potential minimum lower water levels, generated from tidal constant data.

**Figure 5:**
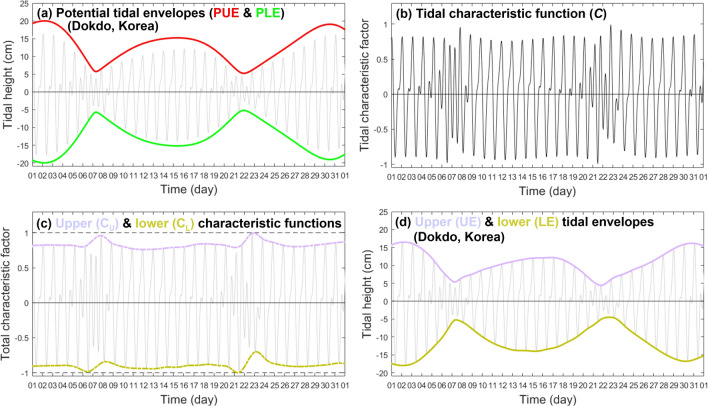
Example procedure for and results of calculating an actual monthly tidal envelope, using sea levels from Dokdo, Korea (August 1 to 31, 2009) and Eqs. (5a), (5b), (6), (7), (8a), and (8b): (**a**) potential maximum upper (PEU, red) and lower (PLE, green) tidal envelopes, compared to actual tidal heights (grey); (**b**) the tidal characteristic function, *C*, that is the ratio between actual tidal heights and PUE; (**c**) tidal characteristic function envelopes, *C*_*U*_ and *C*_*L*_, derived from interpolation of the maximum daily peaks and troughs of *C*; and (**d**) actual upper (UE, purple) and lower (LE, yellow) tidal envelopes, calculated by multiplying PUE by *C*_*U*_ and *C*_*L*_ respectively.

Here we demonstrate a method for predicting the potential maximum upper and minimum lower envelopes as well as the actual upper and lower tidal envelopes, based on a complete tidal species modulation (CTSM) technique. First, conventional tidal harmonic-based prediction approaches may be expressed in CTSM form^17^, using modulated species amplitudes $({A}^{\left(s\right)})$ and phase lags $({\varphi }^{\left(s\right)})$ for each tidal species:

2a $$\eta \left(\uptau \right)=\sum_{k=1}^{N}{f}_{k}{(\tau )a}_{k}\mathrm{cos}\left({\omega }_{k}t+{V}_{k}\left({t}_{0}\right)+{u}_{k}\left(\tau \right)-{g}_{k}\right),$$

2b $$=\sum_{s=1}^{n}{A}^{(s)}\left(\uptau \right)\mathrm{cos}\left({\omega }_{R}^{(s)}t-{\varphi }^{(s)}\left(\uptau \right)\right),$$

with

3 $${A(\tau )}^{(s)}=\sqrt{\sum_{i=1}^{m}{\left[{f}_{i}^{(s)}\left(\tau \right){a}_{i}^{(s)}\right]}^{2}+2\sum_{ i=1 }^{m-2}\sum_{ j=i+1 }^{m}\left[{f}_{i}^{(s)}\left(\tau \right){a}_{i}^{(s)}\right]\left[{f}_{j}^{(s)}\left(\tau \right){a}_{j}^{(s)}\right] \mathrm{cos}\left\{\left({\omega }_{i}^{(s)}-{\omega }_{j}^{(s)}\right)t+\left[{V}_{i}^{(s)}\left({t}_{0}\right)+{u}_{i}^{(s)}\left(\tau \right)-{g}_{i}^{(s)}\right]-\left[{V}_{j}^{(s)}\left({t}_{0}\right)+{u}_{j}^{(s)}\left(\tau \right)-{g}_{j}^{(s)}\right]\right\}},$$

and

4 $${\varphi \left(\uptau \right)}^{(s)}={tan}^{-1}\left(\frac{-\sum_{i=1}^{m}{f}_{i}^{(s)}\left(\tau \right){a}_{i}^{(s)}\mathrm{ sin}[({\omega }_{i}^{(s)}-{\omega }_{R}^{(s)})t+ {V}_{i}^{(s)}\left({t}_{0}\right)+{u\left(\tau \right)}_{i}^{(s)}-{g}_{i}^{(s)}]}{\sum_{i=1}^{m}{f}_{i}^{(s)}\left(\tau \right){a}_{i}^{(s)}\mathrm{cos}[({\omega }_{i}^{(s)}-{\omega }_{R}^{(s)})t+ {V}_{i}^{(s)}\left({t}_{0}\right)+{u}_{i}^{(s)}\left(\tau \right)-{g}_{i}^{(s)}]}\right),$$

for any given time $\tau$ equaling the reference time (${t}_{0}$), plus the time elapsed since ${t}_{0 }(t=\tau -{t}_{0})$, where superscript *s* denotes tidal species (e.g. *s* = 1 is diurnal and *s* = 2 is semidiurnal, etc.), *n* is the number of tidal species, subscript *k* denotes each tidal constituent, subscripts *i* and *j* denote each tidal constituent in tidal species, *N* is the number of tidal constituents, *m* is the number of tidal constituents for each species, ${\omega }_{i}^{(s)}$ is the tidal constituent frequency for each species, ${\omega }_{R}^{(s)}$ is the representative tidal constituent frequency for each species (e.g. ${\omega }_{{K}_{1}}^{(1)}$ and ${\omega }_{{M}_{2}}^{(2)}$ are the diurnal and semidiurnal species representatives of ${\omega }_{R}^{(s)}$), ${a}_{i}^{(s)}$ and ${g}_{i}^{(s)}$ are the amplitude and phase lag of each tidal constituent for each species, and ${V\left({t}_{0}\right)}_{i}^{(s)}$, ${f\left(\tau \right)}_{i}^{(s)}$ and ${u\left(\tau \right)}_{i}^{(s)}$ are the astronomical argument, nodal amplitude factor, and nodal angels of each tidal constituent for each species. Figure 5 illustrates the procedure for calculating actual monthly tidal envelopes, using example sea level data from Dokdo, Korea.

Terms on the right of Eq. (2b) comprise the sum of the amplitude envelope modulation function multiplied by the cosine of the phase modulation function for each tidal species. Assuming the sum of the cosine functions for each species in Eq. (2b) varies from − 1 to + 1, and that constituents within each tidal species are in phase, the sum of the species’ amplitude envelopes yields the potential highest upper and lowest lower tidal envelopes (together the ‘potential maximum tidal envelope’). Accordingly, the PUE and PLE derived from the modulated species amplitudes alone may be expressed as:

5a $$\mathrm{PUE}\left(\uptau \right)=+\sum_{s=1}^{n}{A(\tau )}^{(s)}$$

5b $$\mathrm{PLE}\left(\uptau \right)=-\sum_{s=1}^{n}{A(\tau )}^{(s)}$$

Figure 5a illustrates the PUE and PLE calculated for Dokdo using Eqs. (5a), (5b). Comparisons between actual verses potential maximum tidal envelopes enable us to assess degrees of asymmetry between upper and lower envelope curves. To quantify these differences, Eq. (2b) can be rewritten as two terms consisting of the PEU $\left( {\sum\nolimits_{{s = 1}}^{n} {A\left( \tau \right)^{{\left( s \right)}} } } \right)$ and the normalized tidal variation characteristic function ($C(\tau )$):

6 $$\upeta \left(\tau \right)=\left(\sum_{s=1}^{n}{A\left(\tau \right)}^{\left(s\right)}\right)C\left(\tau \right),$$

and

7 $$C\left(\tau \right)=\frac{\sum_{s=1}^{n}{A\left(\tau \right)}^{\left(s\right)}\mathrm{cos}\left({\omega }_{R}^{\left(s\right)}t-{\varphi \left(\uptau \right)}^{\left(s\right)}\right)}{\sum_{s=1}^{n}{A\left(\tau \right)}^{\left(s\right)}}.$$

As $C(\tau )$ is characterized by normalized tidal heights varying within a ± 1 range, it contains information on relative tidal height variation (Fig. 5b). Further, since the relative maximum and minimum $C(\tau )$ values of each tidal cycle match the timing of the actual high and low waters, these values can be used to determine the degree of asymmetry between upper and lower envelope curves. We extracted these values and, in turn, interpolated the corresponding observation times using MATLAB functions ‘findpeaks’ and ‘spline’ (Fig. 5c) (note the MATLAB function ‘envelope’ can also yield these data). As shown in Fig. 5d, the actual upper ($\mathrm{UE}$) and lower ($\mathrm{LE}$) tidal envelopes can be calculated by multiplying the PUE by the interpolated upper and lower tidal characteristic functions (${C}_{U}\left(\tau \right)$ and ${C}_{L}\left(\tau \right)$) for each time, as given by:

8a $$\mathrm{UE}\left(\uptau \right)=\left(\sum_{s=1}^{n}{A\left(\tau \right)}^{\left(s\right)}\right){C}_{U}(\tau )$$

8b $$\mathrm{LE}\left(\uptau \right)={\left(\sum_{s=1}^{n}{A\left(\tau \right)}^{\left(s\right)}\right)C}_{L}(\tau )$$

Note when the values of ${C}_{U}(\tau )$ and ${C}_{L}\left(\tau \right)$ approach ± 1 at high and low waters respectively, PUE and PLE values correspond closely to the actual tidal heights at high and low waters, such that the potential and actual tidal envelope patterns resemble each other. That is, when ${C}_{U}(\tau )$=1 ${(or C}_{L}(\tau )$=− 1), $\mathrm{UE}\left(\uptau \right)=\mathrm{PUE}\left(\uptau \right)$ in Eq. (5a) (or $\mathrm{LE}\left(\uptau \right)=\mathrm{PLE}\left(\uptau \right)$ in Eq. (5b)).

Since ${C}_{U}(\tau )$ and ${C}_{L}(\tau )$ reflect the characteristics of the actual versus potential maximum and minimum envelopes, these functions may be used as indicators of envelope anomalies. To quantitatively compare tidal envelope characteristics between different sites, we use the mean of the differences between ${C}_{U}(\tau )$ and $\left|{C}_{L}(\tau )\right|$ values over a certain period (e.g., a year), producing a monthly envelope form factor (${E}_{M}$) via:

9 $${E}_{M}=\frac{\sum_{k=1}^{N}{C}_{U}\left({\tau }_{k}\right)-\left|{C}_{L}\left({\tau }_{k}\right)\right|}{N}=\overline{{C}_{U}(\tau )}-\overline{{C}_{L}\left(\tau \right)},$$

with

10 $$\overline{{C}_{U}(\tau )}=\frac{\sum_{k=1}^{N}{C}_{U}\left({\tau }_{k}\right)}{N} \mathrm{and} \overline{{C}_{L}(\tau )}=\frac{\sum_{k=1}^{N}\left|{C}_{L}\left({\tau }_{k}\right)\right|}{N}.$$

The generalized tidal envelope types (Fig. 3) developed using ${E}_{A}$ for a subset of mixed tidal regimes in the previous section can be classified according to ${E}_{M}$ values as follows:$-0.05\le {E}_{M}\le 0.05$ indicates a near-symmetric STE, ${E}_{M}>0.05$ indicates UTE dominance, and ${E}_{M}<-0.05$ indicates LTE dominance. Large absolute ${E}_{M}$ values indicate that the actual tidal envelopes closely resemble their potential maximums, PUE or PLE.

## Evaluation of the new TEF factor approaches

${E}_{M}$ In Eq. (9) was applied to the Table 1 experimental cases 1 to 5, covering each different envelope type distinguished using ${E}_{A}$ while omitting duplicate types. The ${E}_{M}$ values produced are listed alongside those of ${E}_{A}$. Note that unlike for ${E}_{A}$, ${E}_{M}$ results give quantitative indications of the degree of symmetry between upper and lower envelopes, with this method being usable in any mixed tide environment, including ones dominated by shallow water species.

To better understand how $${E}_{M}$$ varies in response to interactions between the semidiurnal and diurnal tides, $${E}_{M}$$ values were calculated for tidal scenarios across a range of different amplitude ratios within the mixed mainly semidiurnal ($$0.25<F<1.5)$$ and mixed mainly diurnal ($$1.5<F<3$$) *F* categories, and for a range of different tidal envelope types categorized according to differences in diurnal phase lag inequalities (types where $${E}_{A}=\pm 1, {E}_{A}=\pm 0.5, {E}_{A}=0$$). For simplicity, we employed the same five tidal constants, and the same diurnal amplitudes listed in Table 1, adjusting the semidiurnal amplitudes to produce different *F* values.

**Figure 6:**
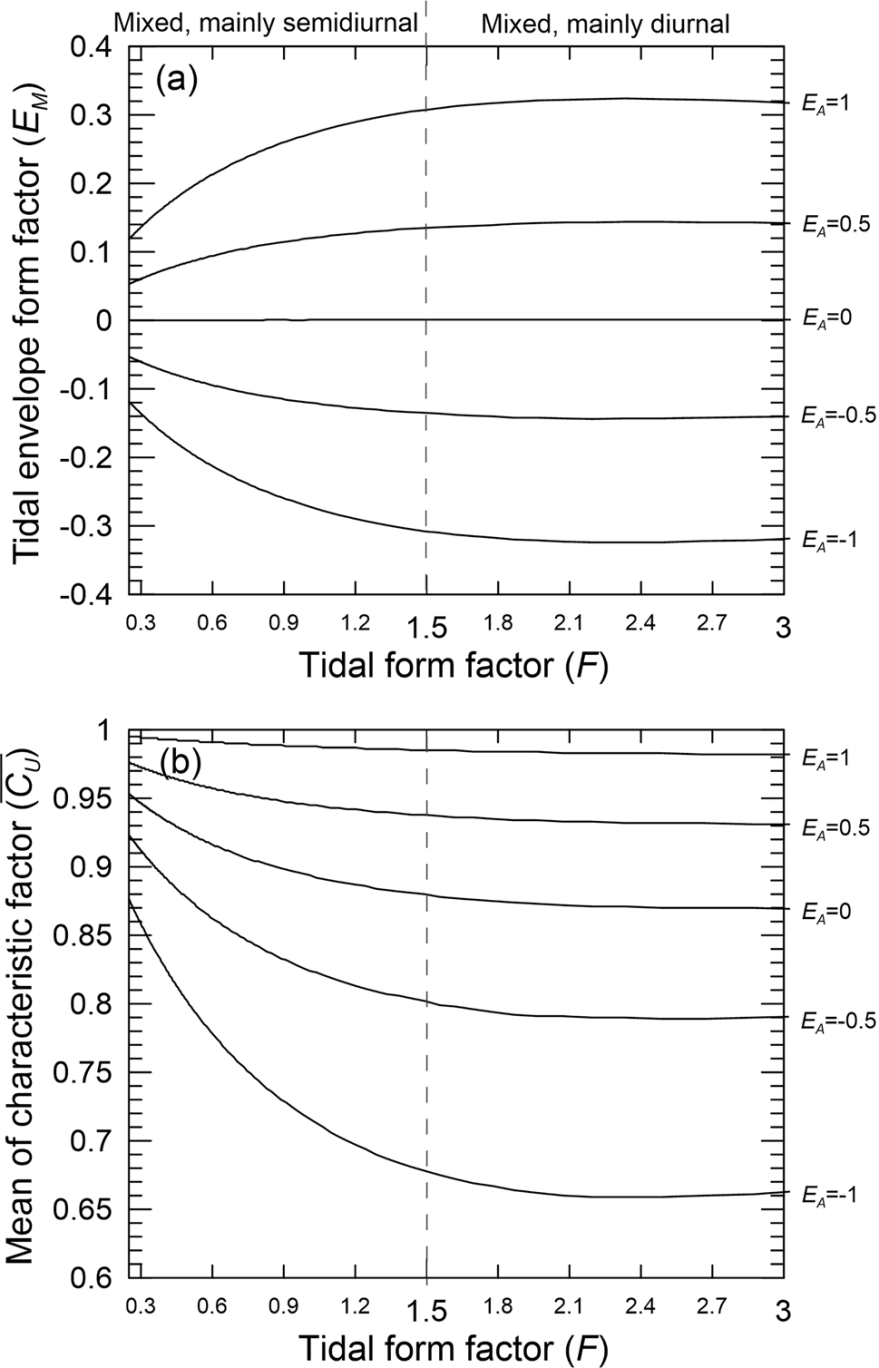
Variations in (**a**) the universal monthly tidal envelope form factor ${E}_{M}$ and (**b**) the mean upper tidal characteristic function $\overline{{C}_{U}}$, corresponding to changes in daily tidal form (*F*) from across the mixed mainly semidiurnal ($0.25<F<1.5$) and mixed mainly diurnal ($1.5<F<3$) regime ranges, for experimental cases where the phase lag differences (${E}_{A}=\mathrm{cos}({I}_{E}$), ${I}_{E}={({g}_{{K}_{1}}+{g}_{{O}_{1}})-g}_{{M}_{2}}$) are ${E}_{A}$ =  ± 1, ± 0.5 and 0. Note that the $\overline{{C}_{U}}$ results for cases where ${E}_{A}=1$,${E}_{A}=-1$, ${E}_{A}=0.5$ and ${E}_{A}=-0.5$ are equivalent to the $\overline{{C}_{L}}$ results (not shown) for cases where ${E}_{A}=-1$, ${E}_{A}=1$, ${E}_{A}=-0.5$ and ${E}_{A}=0.5$, respectively.

Figure 6a reveals that for conditions characterized by ${E}_{A}$ $values >0, {E}_{M}$ values increased significantly with increasing *F* values across the first half of the mixed mainly semidiurnal regimes, increasing more gradually across the second half of these regimes, before plateauing within the range of mixed mainly diurnal tide regimes. For conditions characterized by ${E}_{A}<0, {E}_{M}$ values followed a mirror image (decreasing) pattern.

Across the range of mixed mainly diurnal regimes, ${E}_{M}$ varied by around ± 0.3 between conditions where the diurnal and semidiurnal tides were in phase (${E}_{A}=\pm 1$) to gradually out of phase ($-1<{E}_{A}<1$). This means that for these specific mixed regimes, diurnal to semi-diurnal tide phase lag differences (indicated by ${E}_{A}$) exert greater effects (by up to about 30%) on the symmetry of monthly tidal envelope curves compared to changes in *F*. As such, the phase lag relationship is second only in importance to the magnitude of tidal amplitudes in determining the degree of monthly tidal envelope symmetry in mixed mainly diurnal regimes (Fig. 6a).

Contrastingly, in mixed mainly semidiurnal regimes, both changes in the diurnal to semidiurnal phase lag relationships (indicated by ${E}_{A}$) and changes in *F* influence the degree of asymmetry in monthly tidal envelopes: ${E}_{A}$ variations produced ${E}_{M}$ differences between 0.11 and 0.3, while changes *F* variations produced ${E}_{M}$ differences between 0 and 1.9 (Fig. 6a). Reinforcing this, Fig. 6b reveals similar sensitivities to phase lag differences and *F* values in the mean tidal characteristic function $\overline{{C}_{U}}$ in Eq. (10), a relative measure of the average difference between the potential maximum and actual upper tidal envelopes. Further, when ${E}_{A}=0$ (i.e. STE), $\overline{{C}_{U}}$ > 0.95 at the beginning of the mixed mainly semidiurnal regime range (i.e. 0.25 < F < 0.35, Fig. 6b), indicating that here actual tidal envelopes approximate potential tidal envelopes (i.e. $\overline{{C}_{U}}=1$). In contrast across the whole of the mixed mainly diurnal regimes, $\overline{{C}_{U}}$ is around 0.87 for conditions where ${E}_{A}=0$, indicating a greater difference between actual and potential envelopes for these tides.

These experimental findings are consistent with analyses produced for a globally distributed range of real-world locations (Fig. 4c and Table 2). Year-long monthly tidal envelopes were generated for these stations based on harmonic analysis results from two to three tidal species (i.e. with diurnal and semidiurnal species plus including or excluding the quarterdiurnal species). For Dokdo, the ${E}_{M}$ value generated from two species was − 0.13, with $\overline{{C}_{U}(\tau )}$ = 0.80 and $\overline{{C}_{L}\left(\tau \right)}$ = 0.93, indicating a LTE result. When the quarterdiurnal species was included, the tidal envelope form remained LTE, with ${E}_{M}$ decreasing slightly to − 0.12, $\overline{{C}_{U}\left(\tau \right)}$ = 0.79 and $\overline{{C}_{L}\left(\tau \right)}$ = 0.91. Likewise, for the other non-Korean sites, there was no great difference between the two and three species’ experimental results due to the relatively week quarterdiurnal tidal constituents at these sites (Table 2).

**Table 2:** Comparison of key tidal species’ harmonic analysis and tidal form (*F*) data and our two different tidal envelope classifications (*E*_*A*_ and *E*_*M*_) for ten sites from a range of mixed tide regimes distributed across the globe as derived from sea level observation data.

Tidal observation station locations (& observation periods)	Tidal constants	Key tidal constituents per species							*F* type	${E}_{A}$	${E}_{M}$		Type
		Diurnal		Semidiurnal			Quarter-diurnal				[s = 1 & 2]	[s = 1, 2 & 4]	
		K_1_	O_1_	M_2_	S_2_	N_2_	M_4_	MS_4_					
Dokdo, Korea (8/2009–5/2010)	${a}_{i}^{(s)}$[cm]	5.0	4.8	4.5	1.5	1.2	0.2	0.05	1.63 MMD	− 0.47	− 0.132	− 0.1229	LTE dominant
	${g}_{i}^{(s)}$[°]	349	312	59	78	37	51	271					
Mokpo, Korea (2017)	${a}_{i}^{(s)}$[cm]	30.7	23.9	144.9	49.4	28.6	21.9	16.7	0.28 MMS	0.29	0.031	− 0.064	LTE dominant
	${g}_{i}^{(s)}$[°]	253	218	38	88	16	193	261					
Trieste, Italy (2021)	${a}_{i}^{(s)}$[cm]	18.2	5.4	26.3	15.7	4.3	0.1	0.1	0.56 MMS	− 0.83	− 0.096	− 0.098	LTE dominant
	${g}_{i}^{(s)}$[°]	53	44	242	249	246	270	313					
Vung Tau, Vietnam (2009)	${a}_{i}^{(s)}$[cm]	59.4	44.7	76.7	29.7	16.4	0.9	0.7	0.98 MMS	− 1.00	− 0.264	− 0.263	LTE dominant
	${g}_{i}^{(s)}$[°]	207	166	193	228	172	215	256					
Chabahar, Iran (2015)	${a}_{i}^{(s)}$[cm]	40.0	20.5	66.7	25.7	16.1	0.8	0.5	0.65 MMS	− 0.96	− 0.139	− 0.143	LTE dominant
	${g}_{i}^{(s)}$[°]	207	166	193	228	172	215	256					
Salalah, Oman (2021)	${a}_{i}^{(s)}$[cm]	35.7	18.3	31.4	12.5	8.5	0.2	0.1	1.23 MMS	− 0.99	− 0.225	− 0.221	LTE dominant
	${g}_{i}^{(s)}$[°]	342	345	140	164	129	295	113					
Aden, Yeman (2009)	${a}_{i}^{(s)}$[cm]	40.0	20.3	48.6	21.5	13.6	0.4	0.2	0.86 MMS	− 0.90	− 0.188	− 0.188	LTE dominant
	${g}_{i}^{(s)}$[°]	308	339	245	286	234	187	296					
Brisbane, Australia (2021)	${a}_{i}^{(s)}$[cm]	21.1	11.6	71.7	19.4	13.7	1.4	0.9	0.36 MMS	0.89	0.124	0.136	UTE dominant
	${g}_{i}^{(s)}$[°]	21	352	345	0	342	271	273					
Cilicap, Indonesia (2019)	${a}_{i}^{(s)}$[cm]	19.0	11.8	47.3	24.3	9.5	0.2	0.2	0.43 MMS	0.50	0.071	0.079	UTE dominant
	${g}_{i}^{(s)}$[°]	166	156	24	84	354	25	53					
Colombo, Sri Lanka (2021)	${a}_{i}^{(s)}$[cm]	7.0	3.1	17.8	12.2	2.1	0.4	0.3	0.34 MMS	0.73	0.084	0.083	UTE dominant
	${g}_{i}^{(s)}$[°]	308	339	245	286	234	187	296					

The number of tidal constituents examined per tidal species in the *E*_*M*_ experiments in this study was 21 for the diurnal species, 18 for the semidiurnal species and 7 for the quarterdiurnal species. Dokdo has a mixed, mainly diurnal tide regime while Mokpo, Trieste, Vung Tau, Chabahar, Salalah, Aden, Brisbane, Cilicap and Colombo have mixed, mainly semidiurnal regimes, with Mokpo also being characterized by relatively strong shallow water tides (see Fig. 4c for a location map). Tidal harmonic analyses employed UTide^25^, and data records of between 288 and 365 days per site, with results shown for the 7 most important tidal constituents. ${a}_{i}^{(s)}$ and ${g}_{i}^{(s)}$ indicate the amplitudes and phase lags of the tidal constituents, respectively. *S* indicates tidal species, while the subscript *i* indicates each tidal constituent. *F* is the tidal form number, with MMD indicating mixed, mainly diurnal tides and MMS indicating mixed, mainly semidiurnal tides. ${E}_{A}=cos{{(g}_{{K}_{1}}+{g}_{{O}_{1}}-g}_{{M}_{2}})$, and ${E}_{M}$ is the difference between the mean upper tidal envelope and absolute mean lower tidal envelope (see Eq. (9)). Phase lags for Dokdo and Mokpo were referenced to Korean Standard Time (KST), while for all other sites these were referenced to Greenwich Mean Time. ‘LTE dominant’ denotes an envelope with a lower curve that closely approximates the minimum potential lower tidal envelope curve. ‘UTE dominant’ denotes an envelope with an upper curve that closely approximates the maximum potential upper tidal envelope curve.

In contrast, in the shallow coastal environment of Mokpo, Korea, the $${E}_{M}$$ value calculated based on two species alone was 0.03, with $$\overline{{C}_{U}(\tau )}$$ = 0.95 and $$\overline{{C}_{L}(\tau )}$$ = 0.92, indicating a STE and approaching this site’s potential upper and lower envelopes. However, with the quarterdiurnal species added to calculations, $${E}_{M}$$ shifted to − 0.06, with $$\overline{{C}_{U}(\tau )}$$ = 0.83 and $$\overline{{C}_{L}\left(\tau \right)}$$, = 0.90, indicating LTE dominance. That is, the monthly TEF altered from STE to LTE dominance due to the strong quarterdiurnal shallow water tides with their ebb tide dominance. Note that the amplitude ratio between the semidiurnal M_2_ and quarterdiurnal M_4_ tides ($${a}_{{M}_{4}}/{a}_{{M}_{2}})$$ was 0.15, while their relative phase lag difference ($${2g}_{{M}_{2}}-{g}_{{M}_{4}})$$ was 244°. The ratio of the (M_4_ + MS_4_) to (M_2_ + S_2_) amplitudes was 0.20, large enough to alter the TEF. Clearly shallow water tides such as the quarterdiurnal species can in some particular locations affect tidal envelope symmetry.

**Figure 7:**
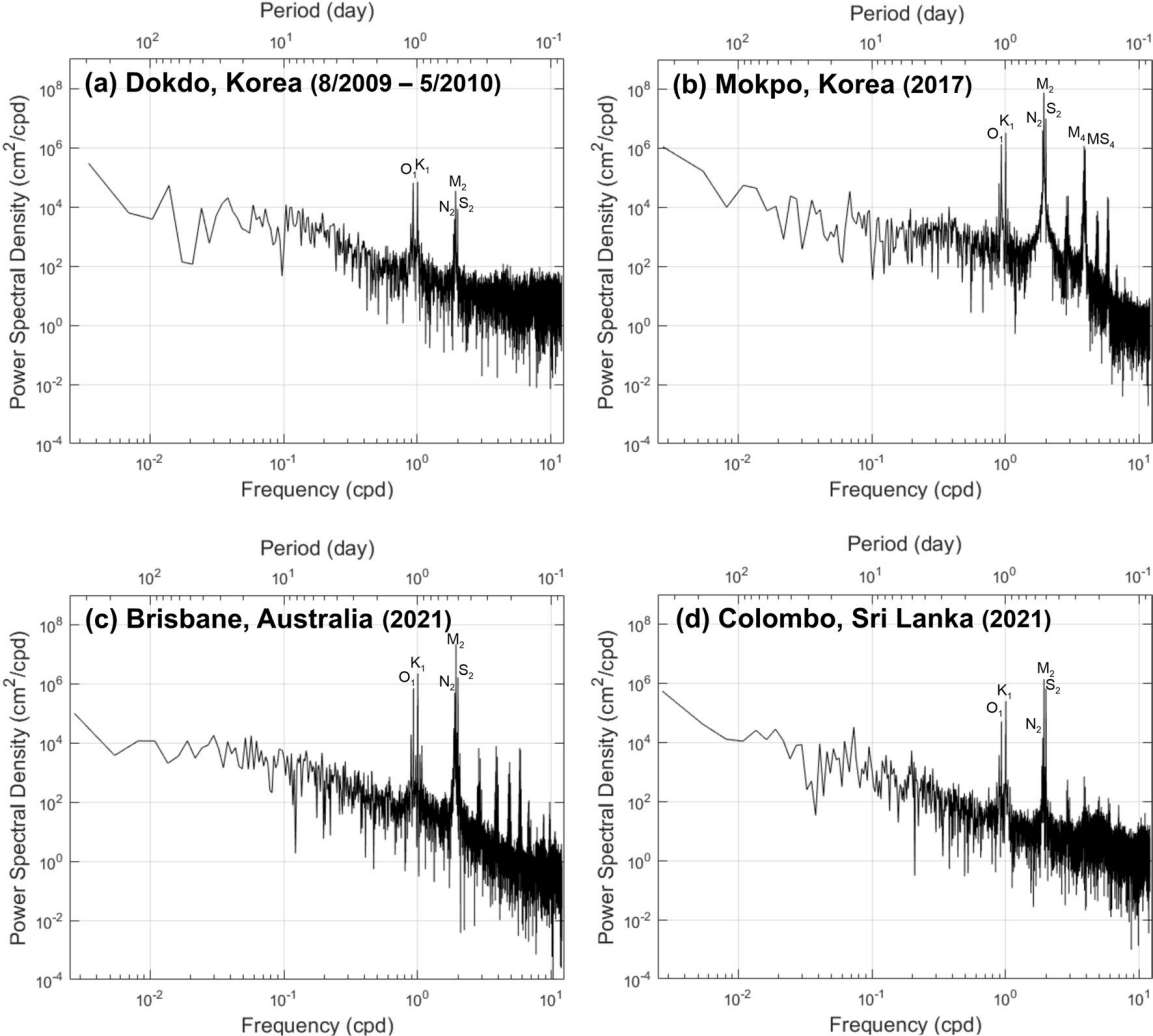
Power spectral density derived from 1 h interval sea level records from (**a**) Dokdo, Korea (August 1 2009 to May 15 2010), (**b**) Mokpo, Korea (2017), (**c**) Brisbane, Australia (2021), and (**d**) Colombo, Sri Lanka (2021). See Ref.^21^ for details of the methods used to generate the power spectra density.

In summary, for most global locations examined in this study, the ${E}_{A}$ and ${E}_{M}$ approaches produced similarly useful results. One exception to this finding was Mokpo, Korea, where the capacity of ${E}_{M}$ to include an additional tidal species changed the tidal envelope classification from STE to LTE dominant. To examine the robustness of the three-species ${E}_{M}$ findings, the Least Squares Spectral Analysis (LSSA) approach known as the Lomb-Scargle periodogram was performed on sea level observations (see details in Table 2) from Mokpo, Dokdo, Brisbane and Colombo, using the method detailed in^21^. The Fig. 7 power spectra results show the dominant tidal species at each site, with Mokpo exhibiting significant peaks for three types of tidal species (diurnal, semidiurnal and quarterdiurnal) whereas the other sites exhibited peaks for only two dominant species (diurnal and semidiurnal). This supports the finding that both ${E}_{A}$ and ${E}_{M}$ can produce robust tidal envelope classifications for sites dominated by two tidal species, but that the application of the ${E}_{M}$ approach using three tidal species can produce more robust results for coastal regimes where three tidal species contribute significantly to the observed water level variations, allowing for the inclusion of significant shallow water tide effects. More work is needed on this approach before a global ${E}_{M}$ distribution can be mapped.

## Conclusion and future research

Mixed tide regimes characteristically have asymmetries between their upper and lower tidal envelopes, such that their potential contribution to coastal inundation varies over medium timescales. Here we have developed two approaches for distinguishing different monthly tidal envelope forms in mixed tide regimes, one extremely simple, qualitative classification with limitations in its spatial applicability, and another more complex, quantitative classification that is universally applicable. The first is a phase lag relationship based monthly TEF factor, ${E}_{A}$ while the second is a tidal species modulation based monthly TEF factor, ${E}_{M}$.

These approaches enable us to classify places and times when the tides are inclined towards higher or lower tidal envelopes than their symmetric potential, with the resulting classifications including upper or lower tidal envelope dominated (UTE and LTE types); or near-symmetric tides (STE). Logically, places characterized by UTE dominance^2,4,5,7,13^ experience increased inundation risk at times each month when the tidal envelope is skewed towards higher high waters and higher low waters. Compared to places with STE forms, this mean increased risk of coastal flooding during 'King tides’ and other extreme events (corresponding to peaks in the upper tidal envelope curve), with extreme floods likely increasing with anthropogenic climate change induced mean sea level rise and increased storminess as well as other weather-related extreme events affecting such coastal locations. Places characterized by LTE dominance^3,8,10,15^, by contrast, are prone to cyclically frequent coastal flooding (corresponding to higher parts of the lower tidal envelope curve) and chronic flooding of low-lying areas today, with this flooding likely to worsen in extent and depth with ongoing climate changes including mean sea level rise and weather pattern change.

With additional development of the ${E}_{M}$ method in particular, our tidal asymmetry classifications could be used as a tidal component indicator in multi-hazard coastal inundation exposure assessments or in hydrological models exploring coastal inundation patterns. Though both classification approaches (${E}_{M}$ and ${E}_{A}$) presented in this paper have arbitrarily determined criteria for STEs—thus we recommend further research to refine the boundaries of this tidal symmetry category. One suggestion is to apply relative entropy ideas to a large tidal dataset, ideas which offer a well-known approach for quantifying statistical distances between probability density functions^22,23^.

## Data Availability

The FES2014 model database of tidal harmonic constants employed in this research is accessible via https://www.aviso.altimetry.fr/en/data/products/auxiliary-products/global-tide-fes.html. Carrère et al.^24^ contains detailed explanations of this model. The sea level observation data used in this work are freely available for download from the Korea Hydrography and Oceanographic Agency (KHOA) online data repository via http://khoa.go.kr/oceangrid/gis/category/reference/distribution.do. 1 h interval sea level records from Brisbane, Cilicap, Trieste, Vung Tau, Chabahar, Salalah, Aden and Colombo tidal stations were obtained from the University of Hawaii Sea Level Center via https://uhslc.soest.hawaii.edu/data/. Tidal harmonic analyses were conducted using UTide Matlab functions^25^, with these functions and a technical report explaining their use available for download from: http://www.po.gso.uri.edu/~codiga/utide/utide.htm. The UTide based CTSM code employed in this study is available from: https://au.mathworks.com/matlabcentral/fileexchange/125690-ctsm_utide.
